# Editorial: Loss of Epithelial Barrier Integrity in Inflammatory Diseases: Cellular Mediators and Therapeutic Targets

**DOI:** 10.3389/fmed.2021.813153

**Published:** 2021-12-09

**Authors:** Carl Weidinger, Susanne M. Krug, Caroline Voskens, Alexander R. Moschen, Imke Atreya

**Affiliations:** ^1^Division of Gastroenterology, Infectious Diseases and Rheumatology, Medical Department, Charité–Universitätsmedizin Berlin, Berlin, Germany; ^2^Clinical Physiology/Nutritional Medicine, Charité-Universitätsmedizin Berlin, Berlin, Germany; ^3^Department of Dermatology, University Hospital Erlangen, Friedrich-Alexander-Universität Erlangen-Nürnberg, Erlangen, Germany; ^4^Deutsches Zentrum Immuntherapie (DZI), University Hospital Erlangen, Erlangen, Germany; ^5^Internal Medicine 2 (Gastroenterology and Hepatology), Faculty of Medicine, Kepler University Hospital, Johannes Kepler University Linz, Linz, Austria; ^6^Department of Medicine 1, University Hospital of Erlangen, Friedrich-Alexander-Universität Erlangen-Nürnberg, Erlangen, Germany

**Keywords:** inflammatory bowel diseases, barrier integrity, immune-epithelial interactions, intestinal mucosa, IBD pathogenesis

## Impaired Intestinal Barrier Integrity In IBD

About 0.75% of the inhabitants of the industrialized countries of the Western world were affected by one of the two main entities of inflammatory bowel diseases (IBD), Crohn's disease or ulcerative colitis, in 2020 (1). This means that a significant part of the general population is affected by chronically remitting gastroenterological symptoms, such as bloody diarrhea, abdominal pain, and anemia. Importantly, besides its high incidence, IBD mainly affects the younger population, who are often significantly hindered in their social and professional lives by the disease. Despite the successful clinical establishment of new hallmark therapies during the last three decades, about 40% of IBD patients do not satisfactorily respond to current treatment strategies or suffer a secondary loss of response (2). Together, this promotes a scientific and clinical interest in identifying innovative therapeutic targets (3), and in this course, there is a steadily growing improvement in knowledge regarding the exact molecular and cellular processes of IBD pathogenesis. Although in the past, many pioneering studies focused on intestinal immune cells (4–6), as their overwhelmingly enhanced activation status and cytokine release were long assumed as predominant drivers of IBD, nowadays, there is growing awareness that the entire mucosal barrier and its function decisively determines the development and resolution of chronic intestinal inflammation (7–9). Addressing this aspect in our Research Topic, we present a diverse collection of original and review articles focusing on the pathological dysregulation and clinical relevance of the mucosal barrier integrity and its cellular key players in IBD.

Besides absorptive and secretory intestinal epithelial cells (IECs) forming the tightly closed epithelial monolayer, a broad spectrum of locally accumulating immune cells including also innate lymphoid cells as a rather newly described immune cell population (reviewed by Schulz-Kuhnt et al.), as well as non-hematopoietic cells, such as endothelial cells, enteric neurons, adipocytes, and fibroblasts, contribute to the protection of the intestinal lamina propria against potentially invading luminal pathogens or foreign antigens (9–16). Furthermore, numerous intestinal blood vessels are located in direct proximity to the epithelial layer and are influenced by local inflammatory processes (e.g., IFNγ-induced disruption of endothelial barrier integrity) (17), while, *vice versa*, the inflammation-triggered increased blood flow supports recruitment of pro-inflammatory immune cells from the circulation (comprehensively summarized by Stürzl et al.). In addition, the enteric nervous system represents another factor of impact on intestinal homeostasis that has been neglected in the past and whose potential contribution in intestinal inflammation and impaired gut barrier is discussed by Drobny et al. While we are constantly getting better understanding of how the dysregulated function of individual cell populations in the gut contributes to the loss of intestinal homeostasis and the initiation or maintenance of chronic inflammatory processes, it is also becoming increasingly clear that the real challenge is deciphering the communication between the different components of this cellular network and to identify central molecular switches driving the loss of mucosal barrier integrity, as well as the counteracting process of mucosal wound healing in IBD (summarized by Sommer et al.). For sure, immune cell-derived cytokines represent central signaling molecules within this and a study by Delbue et al. provides new mechanistic insights into IL-22-mediated effects on epithelial integrity and wound healing. Moreover, implying even higher complexity, the interplay between different cellular compartments of the gut may further be influenced by external factors derived from the lumen, as very well-established for intestinal microbiota (an overview is provided by Jergens et al.) and of clear relevance also for defined nutritional components (18, 19). For example, Yeung et al. observed that reduced uptake of vitamin D resulted in impaired mucosal barrier properties.

## Implications For IBD Therapy

Most of the clinically applied strategies in IBD therapy, including classic immunosuppressive drugs (e.g., azathioprine and 6-mercaptopurine), but also more specific approaches like anti-cytokine antibodies (e.g., anti-TNF therapy and IL-12/IL-23-neutralizing ustekinumab) and anti-adhesion therapy (e.g., vedolizumab) primarily target the pathologically increased activation and/or accumulation of pro-inflammatory immune cells in the intestinal mucosa (3, 6). Lately, the maintenance and restoration of the intestinal barrier function and mucosal healing emerged as relatively new therapeutic goal in the clinical management of IBD (8, 20) and this also resulted in a better consideration of the epithelial-protective effects of established therapeutics. For example, the recognized capacity of anti-TNF therapy to restore the pathologically increased rate of apoptotic IECs and the subsequent loss of epithelial resistance in IBD patients (21), as well as the counteracting influence of azathioprine and 5-aminosalicylic acid on the inflammation-triggered downregulation and rearrangement of junctional proteins in *in vitro* cultured IECs and intestinal organoids (22). In addition, defined molecular mediators and intracellular signaling pathways involved in the maintenance of the epithelial tightness (e.g., STAT6, angulin-1, leptin, RhoA, and IL13Rα2) (23–26), in IEC survival (e.g., Caspase-8) (27), in the production of antimicrobial peptides (e.g., human β-defensin 2) (28) and in wound healing (e.g., STAT1, STAT3) (29, 30) have also been suggested as innovative therapeutic targets. As exemplarily demonstrated by Gerbeth et al. summarizing the multiple effects of histone deacetylase inhibitors on gut homeostasis, it will in general be essential to always consider the above outlined cellular complexity of the protective mucosal barrier and carefully validate the role of potential innovative target structures for the entire panel of involved cell types. Moreover, the fact that pathological conditions significantly differ dependent on the phase of disease (nicely described by Semin et al.) and its site of manifestation (emphasized by the study of Stolzer et al. describing a different impact of STAT1 signaling on IEC cell death in the context of inflammation in ileum and colon, and by comparative transcriptomic results reported by Gonzalez Acera et al.), makes it important to also develop diagnostic strategies allowing a careful clinical and molecular characterization of the individual disease status prior to the selection and initiation of therapy. In this context, Bojarski et al. provide a valuable overview of innovative advanced gastrointestinal endoscopic technologies and their potential future contribution in paving the way for personalized medicine in IBD.

## Author Contributions

All authors listed have made a substantial, direct, and intellectual contribution to the work and approved it for publication.

## Funding

This work has received funding from the DFG, German Research Foundation (SFB TransRegio TRR 241, subprojects A07, B01, B06, and C04).

## Conflict of Interest

The authors declare that the research was conducted in the absence of any commercial or financial relationships that could be construed as a potential conflict of interest.

## Publisher's Note

All claims expressed in this article are solely those of the authors and do not necessarily represent those of their affiliated organizations, or those of the publisher, the editors and the reviewers. Any product that may be evaluated in this article, or claim that may be made by its manufacturer, is not guaranteed or endorsed by the publisher.
